# Therapeutic and protective approaches to combat *Campylobacter jejuni* infections

**DOI:** 10.3389/fphar.2025.1572616

**Published:** 2025-05-06

**Authors:** Irshad Sharafutdinov, Bodo Linz, Nicole Tegtmeyer, Steffen Backert

**Affiliations:** Department of Biology, Division of Microbiology, Universität Erlangen-Nürnberg, Erlangen, Germany

**Keywords:** *Campylobacter* *jejuni*, gut microbiota, epithelial barrier, One Health, phytochemicals, probiotics, bacteriophage, vaccine

## Abstract

*Campylobacter jejuni* is a typical zoonotic bacterium, colonizing the gut of many bird species as commensal. In humans, *C. jejuni* is a major foodborne pathogen. Infection of humans causes campylobacteriosis in the small intestine, constituting a main source of bacteria-dependent gastroenteritis cases worldwide. In particular, the ingestion of under-cooked rooster meat, raw milk and contaminated water, as well as cross-contamination of ready-to-eat food after handling raw chicken meat, are responsible for the majority of *C. jejuni* infections. As a consequence, infected individuals may acquire watery and/or bloody diarrhea associated with abdominal pain, and eventually post-infection illnesses of the neural system and joints, including the Guillain-Barré, Miller Fisher and Reiter syndromes. One therapeutic strategy is to reduce *C. jejuni* colonization in chicken farms using vaccination, bacteriocins and phage therapy protocols. Prevention approaches during poultry meat processing comprise the compliance to high hygiene standards. Furthermore, substantial progress has been also made in recent years to combat campylobacteriosis using established mouse and *in vitro* cell model systems. In this regard, specific *C. jejuni* colonization- and pathogenicity-associated components were considered as favored treatment structures, targeting bacterial movement, host cell interaction, intracellular survival, propagation and spread of the bacteria. This has been complemented by a number of pharmaceutical compounds to reduce *C. jejuni*-induced epithelial cell damage, inflammation and apoptosis in infected mice. Here we review these novel treatment and prevention as well as “One World - One Health” approaches that aim to diminish the consequences of acute campylobacteriosis and post-infection sequelae in humans.

## 1 Introduction

Zoonoses represent major infectious diseases, which are transmitted among animals and humans by bacteria, fungi, viruses, parasites and prions (Plowright et al., 2017; Hasso-Agopsowicz et al., 2021). Around 75% of all infectious diseases are originally transferred from animals to humans, often via contaminated food (Sharan et al., 2023). Annual statistics from the World Health Organization document that approximately 550 million cases of illness are foodborne, including 220 million very young children up to 4 years of age (WHO, 2020). Bacteria-dependent zoonotic diseases in the human intestine are dominated by pathogens of two genera, *Campylobacter* and *Salmonella*. The European Food Safety Authority (EFSA) and the European Centre for Disease Prevention and Control (2024) reported for 16 investigated European countries that campylobacteriosis and salmonellosis are the first and second most common zoonoses of humans, respectively. Although the overall number of *Salmonella* cases reported worldwide has somewhat fallen in recent years, the numbers of *Campylobacter* infections remain constantly high, which causes enormous health problems. For the European Union, the costs of campylobacteriosis were assessed to comprise around 2.4 billion euros annually (EFSA, 2014), while the costs in the U.S. are up to 11.3 billion dollars per year (Hoffmann et al., 2024). Thus, *Campylobacter* is a typical zoonotic and foodborne pathogen that generates an important economic burden in the societies. Species of the genus *Campylobacter* can be regularly found in several natural ecosystems, including surface water and in the gut of birds and various agriculturally important animals (Huang et al., 2015; Mulder et al., 2020; Mughini-Gras et al., 2021). In addition, two predominant *Campylobacter* subspecies (*C. jejuni* and less frequently *C. coli*) can colonize chicken and other farm animals as commensal microbes. Thus, bacterial contamination commonly occurs during slaughter, which always leads to the transfer of *C. jejuni* to meat (Kingsbury et al., 2023). Consequently, the main transmission to humans occurs via the consumption of *C. jejuni-*positive rooster meat products. In addition, unpasteurized milk (Kenyon et al., 2020) and contaminated water (Gilpin et al., 2020; Jansen et al., 2024) were also reported as sources of the infection.

Upon intake by the fecal-oral route, *C. jejuni* passes the upper gastrointestinal tract, followed by colonizing the lower small and large intestines (Bereswill et al., 2011a; Kaakoush et al., 2015). The infectious dose is quite low at a few hundred bacteria. *C. jejuni* then penetrates the intestinal epithelium and triggers acute inflammatory responses by activation of the human innate immune machinery (Lobo de Sá et al., 2021a). Therefore, the course of campylobacteriosis depends on the immune status of the infected person and varies from mild, mildly inflammatory, self-limiting symptoms to severe, vastly inflammatory and blood-containing diarrhoea (Burnham and Hendrixson, 2018; Heimesaat M. M. et al., 2021). These symptoms can be associated with harsh abdominal pain that commonly last for a week or longer. Occasionally, *C. jejuni* infections can also trigger bacteremia and immunologically-mediated post-infectious diseases with an autoimmune component (Backert et al., 2017). These include neural diseases such as the Guillain-Barré and Miller-Fisher syndromes (GBS and MFS), as well as joint inflammation associated with Reiter’s syndrome (RS) or reactive arthritis, which can occur several weeks after *C. jejuni* clearance (Diggikar et al., 2024; Hughes, 2024; Leonhard et al., 2024). In addition, *C. jejuni* infections can also lead to the development of post-infectious irritable bowel syndrome (PI-IBS) and may trigger episodes of chronic inflammatory bowel disease such as ulcerative colitis and Crohn’s disease (Burnham and Hendrixson, 2018; Lobo de Sá et al., 2021a; Heimesaat M. M. et al., 2021). However, the molecular basis of these diseases is still not fully elucidated. Consequently, there is an urgent need for a broader knowledge about the health and disease mechanisms of humans associated with domestic animals and wildlife. To this end, the “One World-One Health” approach is one promising strategy for ensuring better control of zoonotic infectious diseases (One World One Health, 2021; Backert, 2021; Pitt and Gunn, 2024). Furthermore, new drug therapeutics, bacteriophages and vaccines against campylobacteriosis have been under development in recent years. Current strategies also aim to inhibit the inflammatory and cell damage responses by *C. jejuni*, which may be protective against secondary diseases such as GBS, MFS and various chronic inflammatory processes. In this review article, we discuss the current status of anti-*Campylobacter* treatments and recent developments in this important field of human health protection.

### 1.1 *Campylobacter jejuni* virulence factors and interactions with host cells

**FIGURE 1:**
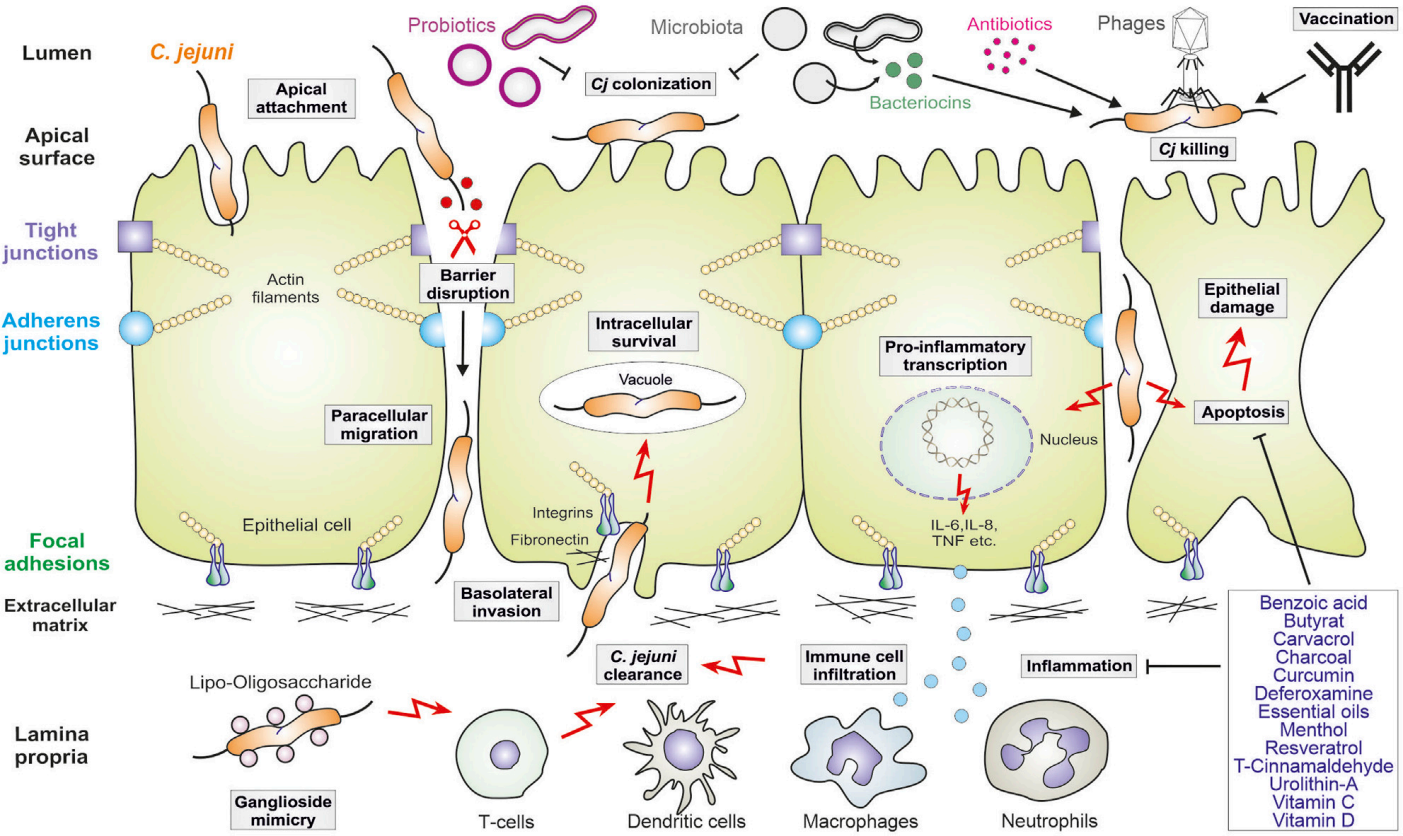
Model for *C. jejuni* virulence mechanisms in human gut infection and therapeutic intervention approaches. The pathogen adheres to polarized intestinal epithelial cells, followed by opening of tight and adherens junctions and basolateral invasion. *C. jejuni* also triggers pro-inflammatory signaling leading to the secretion of cytokines that attract the indicated immune cells, which infiltrate to the site of infection. In this scenario, apoptosis is also induced leading to additional epithelial cell damage. The crosstalk of *C. jejuni* with the intestinal microbiota is indicated. Intervention procedures include the use of probiotics, some of which inhibit *C. jejuni* colonization, as well as bacteriocins, vaccination and phage application. Some antibiotics can be applied, but antibiotic resistances are common in *C. jejuni*. Thus, various indicated drug compounds are new promising therapeutic candidates to inhibit inflammation and apoptosis in the infected intestine. For more details, see the text.

The molecular mechanisms of *C. jejuni* pathogenesis have been investigated using infection of cultured cell lines, various animal model systems and in clinical studies. Campylobacteriosis is characterised by disruption of the intestinal epithelial barrier and inflammation triggered by infiltrating immune cells (model in Figure 1). The invading bacteria move very efficiently with the support of their propelling flagella under the guidance of chemotactic receptors (Korolik, 2019; Cayrou et al., 2021). Afterwards, *C. jejuni* colonizes the mucus layer and attaches to intestinal epithelial cells that is mediated by specific bacterial adhesion factors (Tegtmeyer et al., 2021). Most notably, the adhesins FlpA and CadF have been characterized thoroughly as binding factors of the extracellular matrix protein fibronectin and associated integrin receptors (Konkel et al., 2020). It was reported that intimate attachment of bacteria to the epithelium is ultimately connected with the capability of *C. jejuni* to invade these cells (O Cróinín and Backert, 2012; Konkel et al., 2020; Tegtmeyer et al., 2021). Host cell entry by *C. jejuni* is supported by effector proteins, the *Campylobacter* invasion antigens (such as CiaB and CiaD), which are secreted via a type-III secretion system (T3SS), also encoded by the flagellum (Young G. M. et al., 1999; Konkel et al., 2004; Song et al., 2004; Negretti et al., 2021). Altogether, it was reported that CadF, FlpA and CiaD can cooperatively stimulate host guanine exchange factors (GEFs such as Vav2, Tiam-1, Dock180 and IQGAP1), which activate members of the small Rho GTPases (Rac1 and Cdc42) to trigger actin polymerization and *C. jejuni* invasion (Krause-Gruszczynska et al., 2007; Krause-Gruszczynska et al., 2011; Boehm et al., 2011; Larson et al., 2013; Eucker and Konkel, 2012; Negretti et al., 2021). Remarkably, *C. jejuni* was shown to survive in vacuoles that are formed inside epithelial cells (Watson and Galán, 2008). It appears that *C. jejuni*-containing vacuoles deviate from the canonical endocytosis route that dampens its transport to lysosomes, while uptake of *C. jejuni* by macrophages leads to lysosome formation and subsequently killing of the pathogen (Watson and Galán, 2008).

During these interactions with host cells, *C. jejuni* expose their endotoxin lipooligosaccharide (LOS) to the human immune system, which triggers highly potent inflammatory responses (Figure 1). LOS is a glycolipid consisting of an oligosaccharide moiety and a lipid A core that is recognised by toll-like receptor 4 (TLR4), which activates a cascade of kinases and the pro-inflammatory transcription factor NF-κB (Kuijf et al., 2010; Kløve et al., 2020; Heimesaat M. M. et al., 2021). In response to LOS, dendritic cells and macrophages attract neutrophil granulocytes, which produce reactive oxygen species (ROS) and inflammatory mediators that cause epithelial destruction through the induction of apoptotic cell death (Figure 1). These processes can be intensified by the cytolethal distending toxin, called CDT, which is expressed and secreted by numerous, but not all *C. jejuni* strains (Lara-Tejero and Galán, 2000). Apoptosis results in ulcerative tissue destruction, and the release of pro-inflammatory cytokines and chemokines (including IL-1β, IL-6, IL-8, IL-12, IL-17, IL-22, IL-23, IFN-γ and TNF) intensifies the inflammatory reactions (Heimesaat M. M. et al., 2021; Lobo de Sá et al., 2021a). In addition, activated T cells can produce the cytokines IL-4 and IL-10, which serve as anti-inflammatory factors that counteract severe courses of campylobacteriosis by dampening the immune responses (Heimesaat M. M. et al., 2021). Interestingly, some *C. jejuni* isolates can even increase the inflammatory host response by sialylating their LOS (Kuijf et al., 2010). This could explain the variability of the LOS associated with different disease outcomes upon infection with individual bacterial strains. For example, sialylation of the oligosaccharide portion of LOS enhances binding to and activation of TLR4, thus driving acute inflammation (Kuijf et al., 2010; Stephenson et al., 2013). Finally, sialylated oligosaccharide chains in *C. jejuni* LOS also resemble gangliosides in the human brain. Thus, individual LOS structural variants can induce the production of anti-ganglioside antibodies in the human host, which can subsequently lead to axonal destruction associated with GBS and MFS (Diggikar et al., 2024; Hughes, 2024; Leonhard et al., 2024).

### 1.2 Epithelial barrier disruption by *Campylobacter jejuni* protease HtrA

The cellular lining in every epithelium is a highly organized polarized structure that protects inner tissues from the potentially harmful impact of intruding microbes (Buckley and St Johnston, 2022). Adjacent epithelial cells are connected at their apical sides by proteins in the tight junction (TJ) followed by adherens junction (AJ) that maintain a barrier function and enable selective paracellular diffusion (Zihni et al., 2016). These pivotal cellular junctions that regulate cell motility and molecular signaling via the associated host cytoskeleton were found to be a key target for many bacterial pathogens (Sharafutdinov et al., 2022a). A major *C. jejuni* virulence factor targeting the host TJ and AJ proteins is serine protease HtrA, the structurally active form of which are four trimers assembled into a dodecamer (Zarzecka et al., 2020; Tegtmeyer et al., 2021). *C. jejuni* HtrA, similar to *Helicobacter pylori* HtrA, possesses a hydrophobic amino acid at position 171, which was found to be crucial for protease trimer stability (Zarzecka et al., 2023). To date, the identified host cell substrates for *C. jejuni* HtrA are the tight junction proteins occludin and claudin-8, and the adherens junction protein E-cadherin (Linz et al., 2023), the cleavage sites of which were mapped to the extracellular loop 2 of occludin, to the extracellular loop 1 of claudin-8, and to the extracellular domain 5 of E−cadherin (Boehm et al., 2012; Boehm et al., 2015; Harrer et al., 2019; Sharafutdinov et al., 2020). Cleavage leads to barrier disruption that allows the influx of bacterial antigens and toxins, as well as transmigration of *C. jejuni* to the serosal side (Lobo de Sá et al., 2021a; Heimesaat et al., 2023a). *In vitro* experiments showed that protein cleavage was time-dependent and positively correlated with bacterial transmigration (Sharafutdinov et al., 2024), but opening of the cell junctions also enabled transmigration of commensals such as the intestinal *Escherichia coli* or *Lactococcus lactis* (Sharafutdinov et al., 2022b). Therefore, the HtrA-dependent mechanism can partially explain how microbiota can be translocated across the intestinal epithelia, interact with host immune cells and activate the release of various cytokines upon infection, which can exacerbate disease symptoms. HtrA is released from *C. jejuni* within shed outer membrane vesicles (OMVs) (Elmi et al., 2016). However, recent results showed that the cell junction-damaging proteolytic activity is mediated via HtrA exposed on the bacterial outer membrane upon direct bacterial-host contact (Sharafutdinov et al., 2024).

### 1.3 Animal models of *Campylobacter jejuni* infection

For decades, research on *C. jejuni*-caused campylobacteriosis has been hampered by the lack of suitable animal models. A variety of different animal species have been tested, and some are used to investigate the interaction of humans with the invading *C. jejuni*. However, all of the currently used animal models are associated with their individual problems in that they do not fully reproduce all aspects of *C. jejuni* infection in humans (Mousavi et al., 2021a). In this context, we’d like to quote a truism attributed to George Box: “All models are wrong; some models are useful.” (Box, 1976). Generally, all models represent approximations, that mimic one or several aspects of the pathogen infection and of the associated symptoms observed in humans. Thus, while no model is ever completely accurate, the question is whether it can provide useful information that advances our understanding of the pathogen-host interaction. Several useful animal models for *C. jejuni* infection are discussed below.

#### 1.3.1 Chickens

Chickens and also various wild birds such as ducks, geese and seagulls are natural hosts and frequently colonized by *C. jejuni*. Natural colonization usually follows initial infection with very few bacteria. In broiler chicken farms, stable natural colonization usually occurs after the second or third week of life (Sahin et al., 2003; Cawthraw and Newell, 2010; Hermans et al., 2011), but stable colonization has also been demonstrated in 6-day-old chickens (Connerton et al., 2018). Experimental infection is usually performed by direct administration of a suspension of mid-log growing *C. jejuni* into the crop of chicken chicks. Infection with 10^5^ CFU achieved 100% colonization of the chicken intestine within 48 h (Szott et al., 2020) and resulted in *C. jejuni* loads as high as 10^6^–10^8^ bacterial cells per g intestinal content (Ridley et al., 2008; Szott et al., 2020). Then, high pathogen loads are maintained for weeks, and the birds spread the pathogen with their feces. Possible transmission to poultry breeding and raising facilities is of high concern due to the potential risk for human health. However, despite the high pathogen loads, the immune system of the birds is largely non-responsive to *C. jejuni* colonization, and the avian hosts do not develop overt disease symptoms. The observed absence of disease symptoms has been attributed to an overall low inflammatory responses. Even spread of the bacteria to extra-intestinal organs, including spleen and liver, was reported to not trigger clinical signs of inflammation (Young C. R. et al., 1999; Young et al., 2007; Hermans et al., 2012b). However, recent studies on chickens experimentally infected with *C. jejuni* detected a Th17 inflammatory response that was mediated by IFNγ and IL-17 signaling (Connerton et al., 2018; Chick et al., 2025). The poor avian immune response to *C. jejuni* is connected to the reduced recognition of bacterial LOS. While the *C. jejuni* LOS is a potent activator of TLR4 in humans (Kuijf et al., 2010) as discussed above, it fails to stimulate the avian TLR4 counterpart. Therefore, in contrast to strong acute inflammatory responses seen in humans, the inability of the avian immune system to detect *C. jejuni* causes a benign colonization, which makes *C. jejuni* a commensal in the avian host (Young et al., 2007).

Which genes are necessary for *C. jejuni* colonization? Transposon mutagenesis identified 22 *C. jejuni* genes involved in colonization of the gastrointestinal tract of chickens, several of which were associated with chemotaxis and motility (Hendrixson and DiRita, 2004). The flagellar T3SS was reported to affect pathogenesis in humans via secretion of virulence factors such as CiaA. The flagellar regulatory system also controls expression of FlaA and other flagellar filament proteins, including the so-called flagellar co-expressed determinants (Feds) that were shown to be important for commensal colonization of chickens (Barrero-Tobon and Hendrixson, 2012). Subsequent research revealed that the abundance of short-chain fatty acids (SCFAs) produced by the resident microbiota as well as organic acids modulate the expression of genes involved in *C. jejuni* commensalism in the chicken host (Luethy et al., 2017). Interestingly, *C. jejuni* can discriminate between different intestinal regions based on the concentration of SCFAs and lactate, which allows *C. jejuni* to colonize its preferred location in the intestinal tract. Here, the *C. jejuni* two-component system (TCS) BumSR senses the concentration of the microbiota-generated intestinal metabolite butyrate by its predicted cytoplasmic sensor kinase BumS and regulates transcription of target genes via its transcription activator BumR. Thus, the BumSR TCS is important for the commensal colonization of the avian host (Goodman et al., 2020).

#### 1.3.2 Mammalian hosts

The chicken model is suitable for unraveling and investigating the bacterial factors that are necessary to establish natural colonization of the avian host or to test the ability of vaccines or antimicrobial compounds to prevent colonization with *C. jejuni*. However, the chicken model cannot be used for the analysis of the complex interactions during human *C. jejuni*-caused campylobacteriosis. Other animal models using newborn pigs, weanling ferrets, gnotobiotic canine pups, and primates have several shortcomings as reviewed previously (Fox et al., 1987; Vitovec et al., 1989; Babakhani et al., 1993). Apart from ethical considerations concerning the use of primates, those animal models are associated with high costs and difficulties that require special equipment and trained staff because of handling issues. Due to the limited availability of genetically standardized animals those models do not allow the use of a large number of animals necessary to investigate the pathogen-host interaction in detail (Fox et al., 1987; Vitovec et al., 1989; Babakhani et al., 1993; Backert, 2021). Below we discuss two established mouse models for *C. jejuni* infection that have recently yielded many new and important results.

#### 1.3.3 Mice and the gut microbiota

Mice are highly convenient for the study of bacterial pathogenicity, and they are indeed used in infection models of many different pathogens. The use of mice has several advantages, including ease of handling due to their size, low-cost housing, and their high fertility. Reaching the reproductive age within 2 months and a short gestation period of only 3 weeks allow for three to four generations a year. In addition, mouse lineages are highly inbred so that all offspring of the same sex can be considered genetically identical, which greatly increases reproducibility in animal experiments over the time course of infections and between laboratories. Moreover, the availability of many different genetically modified mouse lineages with altered immune components allows to investigate the roles of each in control and/or clearance of the bacterial infection and disease.

Unfortunately, the mouse model of *C. jejuni* infection is associated with a number of problems. A major drawback is that mice are generally about 500 times more resistant to TLR4 ligands such as LPS and LOS than humans (Vaure and Liu, 2014). Thus, mouse TLR4 is only poorly stimulated by *C. jejuni* LOS, and mice do not react with a robust pro-inflammatory immune response to *C. jejuni* infection (Stahl et al., 2014). In addition, *C. jejuni* only poorly, if at all, colonizes conventional wild-type mice, which led to the term “colonization resistance” in regard to the inability to establish efficient colonization (Jesudason et al., 1989; Dorrell and Wren, 2007; Bereswill et al., 2011b; Masanta et al., 2013). Due to these major complications, work with the mouse model has been neglected for years. Interestingly, in contrast to wild-type mice, stable *C. jejuni* colonization was successfully achieved in germ-free adult mice (Yrios and Balish, 1986a; 1986b; Chang and Miller, 2006). However, germfree mice are known to have an hampered innate immune system (Haag and Siegmund, 2015), which raises the possibility that either the immune answer or the intestinal microbiota or both may be involved in preventing stable *C. jejuni* infection in mice. A major breakthrough in mouse model-based work on campylobacteriosis was achieved after the observation that pre-treatment of mice by oral administration of broad-spectrum antibiotics, which essentially killed the entire intestinal microbiota, enabled stable experimental colonization with *C. jejuni* (Bereswill et al., 2011a), suggesting that the microbiota prevented *C. jejuni* from colonizing mice. Additional evidence came from experiments in which those secondary abiotic mice were orally re-inoculated either with mouse intestinal microbiota or with human-derived intestinal microbiota. These animals, which then permanently carried the human or murine microbiota, were subsequently infected with *C. jejuni* and assessed for bacterial loads of the pathogen. In contrast to secondary abiotic mice and mice carrying human intestinal microbiota, in which *C. jejuni* infection was maintained for 6 weeks, mice re-inoculated with mouse intestinal microbiota cleared *C. jejuni* within 2 days. Thus, the re-introduction of the mouse microbiota re-established the previous colonization resistance, clearly pointing to the microbiota as the cause of the colonization resistance (Bereswill et al., 2011b).

While the possibility to infect those secondary abiotic mice with *C. jejuni* after oral inoculation represented a major step as it overcame the colonization resistance, the problem remained that the infected mice did not display the symptoms known from human campylobacteriosis (Bereswill et al., 2011b; Heimesaat and Bereswill, 2015). Here, the availability of multiple different mouse lineages with altered immune components came to the rescue in the form of mice deficient in IL-10, because IL-10^−/−^ mice developed intestinal immunopathology after oral infection with *C. jejuni* (Mansfield et al., 2007; Mansfield et al., 2008; Bell et al., 2009). Microbiota-depleted IL-10^−/−^ mice could be stably infected with *C. jejuni* and developed an acute enterocolitis with bloody inflammatory diarrhea within 1 week post infection and showed symptoms typical for severe campylobacteriosis in immunocompromised human patients (Haag et al., 2012c; Heimesaat et al., 2014a; Heimesaat et al., 2017; Mousavi et al., 2020a). Likewise, *C. jejuni* infection of secondary abiotic mice lacking single Ig IL-1 receptor-related molecule (SIGIRR) reproduced a typical intestinal pathology. The infected Sigirr^−/−^ mice showed strongly elevated expression of pro-inflammatory cytokines such as TNF, IFN-γ, and IL-17 compared to wild-type mice (Stahl et al., 2014). However, the overall clinical disease symptoms were moderate, and the *C. jejuni* infection was self-limiting (Stahl et al., 2014; Stahl and Vallance, 2015).

The infant mouse model that utilizes conventional 3-weeks-old mice carrying their natural intestinal microbiota avoids some of the above problems and dependencies on altered immune components. When only 3 weeks old, which is immediately after weaning, young mice were found to be more susceptible to *C. jejuni* infection than adult mice (Haag et al., 2012b). Young mice developed self-limiting acute enteritis within 1 week post infection, which was accompanied by inflammation of the colon mucosa and diarrhea with bloody discharge (Haag et al., 2012b; Heimesaat et al., 2014b; Heimesaat et al., 2016), and *C. jejuni* was also detected in extra-intestinal organs. Thus, the infant mouse model of *C. jejuni* infection mimics key features of human campylobacteriosis. The murine gut microbiota are known to change after weaning and to stabilize in their composition in the weeks after (Schloss et al., 2012), and young mice were found to carry more *E. coli* and less *Lactobacillus* in their intestines compared to adult mice (Haag et al., 2012b; Haag et al., 2012a). Subsequent research showed that addition of *E. coli* to the intestinal microbiota of adult mice increased *C. jejuni* loads during infection, effectively mitigating the colonization resistance against *C. jejuni* and suggesting that the proportion of enterobacteria may affect the susceptibility (Haag et al., 2012c). Along those lines, it is interesting to speculate that the relative amount of enterobacteria in the large intestine may also affect the severity of campylobacteriosis in humans, from (almost) asymptomatic carriage via mild diarrhea to severe bloody diarrhea with abdominal cramps.

In general, the development and advancement of novel and modified mouse models of *C. jejuni* infection in the last 2 decades greatly contributed to our understanding of *C. jejuni* interactions with the human host and the associated pathogenicity. However, despite the exciting progress that has been made, the mouse models of *C. jejuni* infection are still far from ideal. In order to enable *C. jejuni* infection, infant mice are used or adult mice are subjected to a 10-week course of broad-spectrum antibiotic treatment administered via the drinking water (Bereswill et al., 2011a). Those secondary abiotic mice are then orally inoculated twice with 10^9^ CFU of mid-log-phase growing bacteria, usually on two consecutive days, to establish stable infection (Schmidt et al., 2019a; Schmidt et al., 2019b). In contrast, infection of the human host only requires oral uptake of a very limited number of bacteria, likely only a few hundred, to establish initial colonization of the intestinal mucus layer (Heimesaat M. M. et al., 2020).

### 1.4 Therapeutics and prevention strategies

The most common transmission route of *C. jejuni* to humans occurs through contaminated food, with the poultry being a major source followed by cattle (Ahmed and Gulhan, 2022). Complex biocontrol of the food processing at all stages, from harvesting and slaughter to the storage and transport to consumers, might help to reduce the burdens of *C. jejuni* contamination (Alter and Reich, 2021; Taha-Abdelaziz et al., 2023). Additionally, *Campylobacter* emission into environment that is responsible for the bacterial persistence, can be significantly reduced by simultaneous application of various intervention strategies (Szott and Friese, 2021). *C. jejuni* infections usually do not require medical treatment, however, in severe cases antibiotic treatment is recommended (Zainol et al., 2024). As a result of improper antibiotic use both in humans and animals, the global growth of antibiotic resistance imposes high risk of common antibiotics to become ineffective against *Campylobacter* infections (Barata et al., 2024). Substantial research has been performed to identify alternative prevention and treatment strategies, as summarized in Table 1 and discussed below. Most of the therapeutic compounds that we summarized below are natural substances produced by plants or bacteria. The majority of these compounds was only recently tested in their ability to inhibit growth of *C. jejuni*, mostly in mouse experiments, some in cell lines *in vitro*. As the majority of those papers were published very recently, there are almost no data yet concerning their application in humans. Therefore, we listed those potential therapeutic compounds as promising.

**TABLE 1:** Therapeutic and protective compounds to combat *Campylobacter* infections.

	Compounds	Tested model system	Results	Clinical outcome (Score)^a^	References
Mouse experiments	Benzoic acid	IL-10^−/−^ knockout mice	Less campylobacteriosis, less inflammation, reduced epithelial cell apoptosis, no effect on *C. jejuni* colonization loads	Benzoic acid-treated group: 7 Placebo group: 10	Du et al. (2023), Du et al. (2024)
	Butyrate	IL-10^−/−^ knockout mice	Less campylobacteriosis, less inflammation, reduced epithelial cell apoptosis, no effect on *C. jejuni* colonization loads	Butyrate group: 8 Placebo group: 10	Du et al. (2022)
	Carvacrol	IL-10^−/−^ knockout mice	Reduced enterocolitis, less inflammation, reduced epithelial cell apoptosis, up to 2 orders of magnitude reduction in intestinal *C. jejuni* loads	Carvacrol group: 2 to 4 Placebo group: 10	Mousavi et al. (2020b), Mousavi et al. (2023a)
	Carvacrol	IL-10^−/−^ knockout mice	Less campylobacteriosis, less inflammation, reduced epithelial cell apoptosis in mice with human gut microbiota (hma mice), up to 2 orders of magnitude reduction in intestinal *C. jejuni* loads	Carvacrol group: 3 to 4 Placebo group: 5 to 10	Foote et al. (2023), Heimesaat et al. (2024a), Mousavi et al. (2023a)
	Charcoal (activated)	IL-10^−/−^ knockout mice	Less campylobacteriosis, less epithelial cell apoptosis, less inflammation, up to 1 order of magnitude reduction in intestinal *C. jejuni* loads	Charcoal group: 5 Placebo group: 10	Bereswill et al. (2021b)
	Charcoal (activated)	IL-10^−/−^ knockout mice	Less campylobacteriosis, less inflammation, less epithelial cell apoptosis in hma mice, no effect on *C. jejuni* colonization loads	Charcoal group: 0 Placebo group: 3	Heimesaat et al. (2024d)
	*Trans*-cinnamaldehyde	IL-10^−/−^ knockout mice	Reduced inflammation, reduced epithelial cell apoptosis, no effect on *C. jejuni* colonization loads	Trans-cinnamaldehyde group: 7 Placebo group: 10	Heimesaat et al. (2023b)
	Curcumin	IL-10^−/−^ knockout mice	Reduced inflammation, reduced epithelial cell apoptosis, up to 1 order of magnitude reduction in intestinal *C. jejuni* loads	Curcumin group: 3 Placebo group: 10	Heimesaat et al. (2024c)
	Deferoxamine	IL-10^−/−^ knockout mice	Reduced inflammation, reduced epithelial cell apoptosis, no effect on *C. jejuni* colonization loads	Deferoxamine group: 5 Placebo group: 8	Bereswill et al. (2023)
	Essential oils	IL-10^−/−^ knockout mice	Less campylobacteriosis, less inflammation, less epithelial cell apoptosis, up to 2 order of magnitude reduction in intestinal *C. jejuni* loads	Essential oils group: 3 to 6 Placebo group: 8 to 11	Bereswill et al. (2021a), Heimesaat et al. (2021b), c; Mousavi et al. (2021b), 2023b
	Menthol	IL-10^−/−^ knockout mice	Less campylobacteriosis, less inflammation, no effect on *C. jejuni* colonization loads	Menthol group: 5 Placebo group: 10	Bandick et al. (2023)
	Menthol	IL-10^−/−^ knockout mice	Less *Campylobacter* colonization, less inflammation, less epithelial cell apoptosis, up to 2 orders of magnitude reduction in intestinal *C. jejuni* loads	Menthol group: 4 Placebo group: 9	Heimesaat et al. (2024b)
Mouse experiments	Probiotics	C57BL/6j mice	Reduction of pro-inflammatory response, stimulation of anti-inflammatory immune responses, no effect on *C. jejuni* colonization loads	Not applicable	Ekmekciu et al. (2017)
	Probiotics	IL-10^−/−^ knockout mice	Reduced inflammation, reduced epithelial cell apoptosis, no effect on *C. jejuni* colonization loads	Probiotics group: 5 Placebo group: 8	Bereswill et al. (2017), Heimesaat et al. (2021d)
	Resveratrol	IL-10^−/−^ knockout mice	Less cell apoptosis, reduced inflammation, less barrier dysfunction in hma mice, up to 1 order of magnitude reduction in intestinal *C. jejuni* loads	Resveratrol group: 4 Placebo group: 12	Heimesaat et al. (2020b)
	Resveratrol	IL-10^−/−^ knockout mice	Reduced campylobacteriosis, less inflammation and epithelial cell apoptosis in hma mice, no effect on *C. jejuni* colonization loads	Resveratrol group: 1 Placebo group: 3	Heimesaat et al. (2023c)
	Urolithin-A	IL-10^−/−^ knockout mice	Less inflammation, less epithelial cell apoptosis, up to 1 order of magnitude reduction in intestinal *C. jejuni* loads	Urolithin-A group: 4 Placebo group: 11	Mousavi et al. (2021c)
	Vitamin C	IL-10^−/−^ knockout mice	Reduced inflammation, less epithelial cell apoptosis, no effect on *C. jejuni* colonization loads	Vitamin C group: 2 Placebo group: 10	Mousavi et al. (2019a)
	Vitamin D	IL-10^−/−^ knockout mice	Less campylobacteriosis, less inflammation, reduced epithelial cell apoptosis, no effect on *C. jejuni* colonization loads	Vitamin D group: 10 Placebo group: 10	Mousavi et al. (2019b), Lobo de Sá et al. (2021c)
Chicken	Bacteriocins	Chicken at farms	Up to 8 order of magnitude reduction of *Campylobacter* colonization levels	Not applicable	Svetoch et al. (2005), Line et al. (2008)
	Bacteriophages	Chicken at farms	Up to 3 order of magnitude reduction of *Campylobacter* colonization levels	Not applicable	Wagenaar et al. (2005), Kittler et al. (2021)
	Chlorinated water	Chicken at slaughter	Up to 1 order of magnitude reduction of *Campylobacter* load on carcasses	Not applicable	Northcutt et al. (2005), Northcutt et al. (2007)
	Probiotics	Chicken at farms	Up to 6 order of magnitude reduction of *Campylobacter* colonization levels	Not applicable	Balta et al. (2022)
	Vaccination	Chicken at farms	Up to 7 order of magnitude reduction of *Campylobacter* colonization levels	Not applicable	Neal-McKinney et al., 2014; Meunier et al., 2016; Nothaft et al., 2016
*in vitro* cell infection	Carvacrol	Caco-2 cell line	Reduction of *C. jejuni* attachment, invasion and translocation	Not applicable	Upadhyay et al. (2017)
	*Trans*-cinnamaldehyde	Caco-2 cell line	Reduction of *C. jejuni* attachment, invasion and translocation	Not applicable	Upadhyay et al. (2017)
	Resveratrol	HT-29/B6 cell line	Prevention of *C. jejuni*-induced barrier dysfunction	Not applicable	Lobo de Sá et al. (2021b)
	Vitamin D	HT-29/B6 cell line	Reduced *C. jejuni* attachment, invasion, cytotoxicity, *C. jejuni*-induced barrier dysfunction and transmigration	Not applicable	Bücker et al. (2018), Lobo de Sá et al. (2021c)

^a^ The clinical outcome at 6 days post infection, with a maximum score of 12 points. Assessment of fecal blood, diarrhea, and other clinical symptoms, including wasting. 12 points: severe symptoms, 0 points: healthy condition. Scoring details as in Heimesaat et al. (2014a).

#### 1.4.1 Antibiotics

While in most infected individuals the *C. jejuni* infection is self-limiting, usually no specific treatment is needed for mild and even moderate symptoms, but is recommended, however, for severe manifestations (Zainol et al., 2024). The European Centre for Disease Prevention and Control (ECDC) reported 137,107 confirmed cases of campylobacteriosis in 30 European countries in 2022. Of the 44,876 cases with a recorded hospitalization status (hospitalized or not), 10,551 (23.5%) were treated at hospitals (European Centre for Disease Prevention and Control, 2024). Likewise, in 2023, 148,181 campylobacteriosis cases were reported in the European Union with 12,194 cases (23.9%) requiring hospitalizations (European Food Safety Authority European Centre for Disease Prevention and Control, 2024). Based on a large combined case-control and source attribution study, a substantial proportion (31%) of human *Campylobacter* infections were treated with antibiotics (Rosner et al., 2017). However, the use of antibiotics may be difficult in controlling *C. jejuni*-caused diarrhea, because *C. jejuni* developed resistance against several antibiotics that have been widely used in veterinary medicine since the 1990s, including tetracycline (Wozniak-Biel et al., 2017), fluoroquinolones (Nachamkin et al., 2002), macrolides (Gibreel et al., 2005), and others (Veronese and Dodi, 2024). Inappropriate use of antibiotics promotes the growing spread of antibiotic resistance, which makes available treatments increasingly ineffective (Barata et al., 2024). As such, selection pressure needs to be removed from the source, so that antibiotic resistance does not confer a competitive advantage to resistant isolates. However, as a recent study from the Netherlands suggests, “reducing antimicrobial use in livestock alone may not be sufficient to reduce antimicrobial resistance among human *Campylobacter* infections” (Deng et al., 2024). An alarmingly increasing number of isolates already exhibits multidrug resistance, including *C. jejuni* from Portugal (Santos-Ferreira et al., 2022) and Brazil (Dias et al., 2021), but most dramatically in Asia, where the proportion of multidrug resistant isolates is close to 100% (Shin et al., 2012; Thomrongsuwannakij et al., 2017). Azithromycin is usually the first-line treatment for *C. jejuni* infections, particularly in cases where symptoms are severe or the patient is immunocompromised. Depending on the geographic area and guidelines, antibiotics of second choice might include fluoroquinolones, rifaximin and trimethoprim-sulfamethoxazole (Veronese and Dodi, 2024). However, due to high (33.1%) to extremely high (100%) resistance levels in Europe, fluoroquinolones cannot be recommended for the treatment of *Campylobacter* infections in humans (European Food Safety Authority & European Centre for Disease Prevention and Control, 2024). Interestingly, experimental depletion of the microbiome in mice by the combination of cefoperazone/sulbactam, as discussed above, resulted in long-term colonization of *C. jejuni* in the intestine (Chen et al., 2024). Another study showed that 8-week long pretreatment by either combination of ampicillin/sulbactam or the antibiotic cocktail (ampicillin, ciprofloxacin, imipenem, metronidazole, and vancomycin) effectively depleted the microbiota in IL-10^−/−^ mice enabling sufficient *C. jejuni* colonization (Heimesaat et al., 2022). These data suggest, that in case of resistance antibiotics therapy may backfire and exacerbate *C. jejuni* colonization. Also, the campylobacteriosis-specific nature of disease associated with dehydration of the organism complicates the control of antibiotic concentrations at the infection site (Heimesaat M. M. et al., 2021). Therefore, alternative treatment strategies especially in resistant *C. jejuni* infections are urgently needed.

#### 1.4.2 Benzoic acid

Naturally present in plants and animals, benzoic acid and its derivatives are known as food supplements for preservation and/or flavoring purposes. For example, salts of benzoic acid (especially sodium benzoate) can exhibit antimicrobial properties via bacterial membrane disruption (del Olmo et al., 2017). Supplementing broiler chickens with benzoic acid led to a decrease in gut pH, which might be beneficial for a healthy microflora, and upregulated gene expression of the junction proteins E-cadherin and claudin-1, which contributes to restoring the integrity of the epithelial barrier (Yu et al., 2024). Antibacterial susceptibility testing on *C. jejuni* isolates *in vitro* showed antimicrobial activity of benzoic acid alone and exhibited synergistic effects in combination with other organic acids, e.g., sorbic acid, propionic acid or acetic acid (Peh et al., 2020). However, *in vivo* infection of IL-10^−/−^ mice showed no antimicrobial activity of benzoic acid alone, and only in combination with butyric, caprylic and sorbic acids *C. jejuni* loads were reduced in the duodenum, but not in the stomach, ileum or colon (Du et al., 2023). Interestingly, treatment with these organic acids improved overall clinical outcome with reduced diarrheal symptoms in *C. jejuni* infected IL-10^−/−^ mice, reduced inflammatory immune responses and dampened secretion of pro-inflammatory cytokines in the colon and mesenteric lymph nodes and the serum (Du et al., 2023). Treatment with benzoic acid alone, similarly to the combination of organic acids, reduced the diarrheal symptoms, alleviated apoptotic cell responses and reduced pro-inflammatory immune reactions (Du et al., 2024). Therefore, benzoic acid appears a promising non-antibiotic immune-modulatory treatment against *C. jejuni* infections.

#### 1.4.3 Butyrate

Butyrate is a salt of butyric acid, which is physiologically produced by intestinal bacteria, is important for the function of immune system, energy metabolism and even the nervous system (Du et al., 2021). *In vitro* studies revealed antimicrobial activity of butyric acid against Gram-negative pathogens *Staphylococcus pseudintermedius* and *Acinetobacter baumannii* through affecting the bacterial membrane integrity (Kennedy et al., 2019). Treatment of *C. jejuni*-infected IL-10^−/−^ mice with butyrate led to reduced diarrheal severity and frequency (Du et al., 2022). Furthermore, butyrate treatment of infected mice significantly reduced intestinal pro-inflammatory immune responses as shown by lower numbers of apoptotic cells and neutrophils, less distinct TNF secretion in mesenteric lymph nodes and lower IL-6 and MCP-1 concentrations in the ileum (Du et al., 2022). Therefore, the above studies indicate that butyrate is a promising molecule for the development of new strategies to combat acute campylobacteriosis.

#### 1.4.4 Activated charcoal

Activated charcoal is an adsorbent with exceptionally few side effects and widely used in management of diarrhea to clear the intoxicating particles from the body (Senderovich and Vierhout, 2018). Treatment of *C. jejuni*-infected IL-10^−/−^ mice with activated charcoal resulted in lower intestinal pathogen loads as well as alleviated clinical signs of diarrhea and diminished pro-inflammatory immune responses in the intestinal tract of mice (Bereswill et al., 2021b). When activated charcoal was used for the treatment of the *C. jejuni* infected human gut microbiota associated (hma) IL-10^−/−^ mice, the campylobacteriosis symptoms and pro-inflammatory signaling were significantly reduced (Heimesaat et al., 2024d). Interestingly, activated charcoal treatment did not affect the fecal microbiota composition that was shifted towards higher enterobacterial numbers and lower loads of obligate anaerobic species in hma IL-10^−/−^ mice (Heimesaat et al., 2024d). Therefore, the group proposed that activated charcoal might be a promising antibiotics-independent option for the treatment of human campylobacteriosis.

#### 1.4.5 Deferoxamine

Deferoxamine is an iron chelator and siderophore that is naturally present in the bacterium *Streptomyces pilosus*, and as a medication used to treat patients with the acute or chronic iron overload (Rahimzadeh et al., 2023). In turn, iron acquisition plays an important role not only for *Campylobacter* survival, but also for effective host colonization (Miller et al., 2009). Therefore, limiting free iron using additional iron chelators such as deferoxamine might be an effective strategy against bacterial colonization. Deferoxamine prophylactically given to microbiota-depleted IL-10^−/−^ mice 7 days prior *C. jejuni* infection did not affect bacterial loads in the mice intestine, but significantly improved the clinical outcome of infected mice at day six post-infection (Bereswill et al., 2023). The pre-treatment with deferoxamine resulted in less epithelial cell apoptosis and reduced intestinal and systemic immune responses in IL-10^−/−^ mice (Bereswill et al., 2023). Furthermore, application of deferoxamine in *C. jejuni*-infected hma IL-10^−/−^ mice at 2 days post infection, diminished recruitment of colonic neutrophils and T lymphocytes and inhibited *ex-vivo* IFN-γ secretion in the colon, the kidneys and in the serum (Mousavi et al., 2024). Interestingly, non-covalent fusion of deferoxamine with chitosan and iron (III) sharply potentiated its anti-bacterial activity against *Staphylococcus aureus* and *E. coli* as shown *in vitro* and *in vivo* using rat peritonitis infection model (Khubiev et al., 2023). Therefore, it would be interesting to test such promising deferoxamine derivatives in campylobacteriosis animal models, which might offer a new non-antibiotic therapeutic strategy.

#### 1.4.6 Phytochemicals

Phytochemicals, which comprise plant-derived antimicrobial agents including extracts, essential oils and purified compounds, have been extensively studied for their antimicrobial potential (Alvarez-Martínez et al., 2021; Balta et al., 2021c). They have been reported to exhibit different antimicrobial mechanisms, most of which are based on disruption of the bacterial membrane. Because the *Campylobacter* infection site is normally limited to the gastrointestinal tract, the application of natural plans-based treatments, that could be orally administered, represents a promising treatment strategy (Alvarez-Martínez et al., 2021).

##### 1.4.6.1 Carvacrol

Carvacrol is a phenolic monoterpene naturally found in essential oils obtained from various plants such as oregano, bergamot and thyme, and which shows promising antimicrobial and antioxidant properties (Maczka et al., 2023). *In vitro* treatment of Caco-2 cells with carvacrol significantly reduced attachment, invasion and translocation of *C. jejuni* (Upadhyay et al., 2017). Four-day carvacrol diet in secondary abiotic IL-10^−/−^ mice prior *C. jejuni* infection reduced pathogen loads in their intestines at 6 days post-infection, and furthermore significantly alleviated acute enterocolitis symptoms (Mousavi et al., 2020b). In addition, carvacrol-fed mice showed decreased intestinal apoptosis rate and diminished pro-inflammatory signaling as evidenced by lower IFN-γ, TNF, MCP-1 and IL-6 concentrations in the intestine (Mousavi et al., 2020b). Importantly, carvacrol treatment restricted *C. jejuni* translocation from the intestine to extra-intestinal compartments of mice, suggesting positive effect of carvacrol on the intestinal barrier. Carvacrol in combination with butyrate, ellagic acid and 20-fucosyl-lactose showed even more pronounced anti-inflammatory effects in *C. jejuni* infected mice by diminishing IFN-γ, TNF, and IL-6 secretion in colon, liver, kidneys, lungs, and serum samples (Mousavi et al., 2023a). In order to simulate the human gut microbiota, secondary abiotic IL-10^−/−^ mice were subjected to oral transplantation of fecal microbiota from human donors (Foote et al., 2023). The hma IL-10^−/−^ mice were further infected with *C. jejuni* and treated with carvacrol, which eventually dampened intestinal and extra-intestinal inflammatory responses during acute campylobacteriosis (Foote et al., 2023). Interestingly, prophylactical pretreatment of hma IL-10^−/−^ mice with carvacrol a week before *C. jejuni* infection significantly mitigated acute campylobacteriosis without affecting the fecal gut microbiota composition (Heimesaat et al., 2024a). Treatment of hma IL-10^−/−^ mice after *C. jejuni* infection with carvacrol alone or in combination with deferoxamine, deoxycholic acid and 2-fucosyl-lactose showed comparable results with diminished immune response in the colon, kidneys and serum, establishing carvacrol as effective anti-*C. jejuni* compound (Mousavi et al., 2024). In chickens, a diet supplemented with carvacrol resulted in changes in the gut microbiome, with an increasing relative abundance of *Lactobacillus* and reduction of *Campylobacter spp*. (Kelly et al., 2017).

##### 1.4.6.2 *Trans*-cinnamaldehyde

*Trans*-cinnamaldehyde is an aromatic aldehyde naturally found in the bark of cinnamon tree and has demonstrated significant antimicrobial activity via disruption of the bacterial cell membrane (Jimenez et al., 2024). *In vitro* studies showed that *trans*-cinnamaldehyde can effectively reduce *C. jejuni* attachment, invasion and translocation in Caco-2 cells (Upadhyay et al., 2017). Prophylactic treatment of IL-10^−/−^ mice with *trans*-cinnamaldehyde starting 7 days prior *C. jejuni* infection did not affect the bacterial colonization but alleviated colonic epithelial cell apoptosis and resulted in less pronounced pro-inflammatory responses (Heimesaat et al., 2023b). The immune-modulatory effects upon *trans*-cinnamaldehyde treatment were not restricted to the intestinal tract of IL-10^−/−^ mice and were also observed in the liver and kidneys (Heimesaat et al., 2023b). The above studies identify *trans*-cinnamaldehyde as a promising prophylactic option for future studies to combat *C. jejuni* infections.

##### 1.4.6.3 Curcumin

Curcumin is a polyphenol produced by plants of the *Curcuma longa* species and exhibits a wide variety of biological activities including antimicrobial, antioxidant, anti-inflammatory, anticancer and many others (Sharifi-Rad et al., 2020). Food supplementation of secondary abiotic IL-10^−/−^ mice with curcumin 1 week prior *C. jejuni* infection resulted in less pronounced histopathological changes and epithelial cell apoptosis in the colon (Heimesaat et al., 2024c). Furthermore, curcumin pretreatment reduced pro-inflammatory innate and adaptive immune responses in *C. jejuni*-infected mice (Heimesaat et al., 2024c). Given its food safety and effectiveness, curcumin warrants further studies for potential application of this polyphenolic compound for the prevention of campylobacteriosis and related post-infectious complications.

##### 1.4.6.4 Menthol

Menthol is a cyclic monoterpene found within essential oils obtained from plants of the *Mentha* genus, with pronounced anti-bacterial and anti-inflammatory activities (Kamatou et al., 2013). Treatment of *C. jejuni*-infected microbiota-depleted IL-10^−/−^ mice with menthol from day two until day six post-infection did not affect the pathogen loads, but reduced the number of colonic T cells (Bandick et al., 2023). Furthermore, application of menthol in combination with the plant-derived extracts used in herbal medicine, alleviated *C. jejuni*-induced diarrheal symptoms in infected mice (Bandick et al., 2023). Interestingly, when synthetic menthol was given to hma IL-10^−/−^ mice prophylactically starting 1 week before *C. jejuni* infection, bacterial loads and immune cell recruitment in the colon were reduced with minimal effects on the human fecal gut microbiota (Heimesaat et al., 2024b). The discussed preclinical placebo-controlled intervention studies indicate on a promising potential of the menthol application against acute campylobacteriosis as an alternative non-antibiotic treatment strategy.

##### 1.4.6.5 Resveratrol

Resveratrol is a polyphenolic molecule naturally found in grapes and therefore present in wines, and in some other plants. Resveratrol plays a protective role against various microbial pathogens (Keylor et al., 2015). Resveratrol treatment of *C. jejuni*-infected secondary abiotic IL-10^−/−^ mice improved the clinical conditions, reduced colonic epithelial apoptosis and dampened innate and adaptive immune cell responses (Heimesaat M. et al., 2020). Importantly, resveratrol treatment could effectively rescue colonic epithelial barrier function in *C. jejuni* infected mice as shown by the transmural electrical resistance assay (Heimesaat M. et al., 2020). Mechanistically, *C. jejuni*-infection disrupted the epithelial barrier in human colonic HT-29/B6 cell monolayers via mis-localization of the TJ proteins occludin and claudin-5, which was restored after resveratrol treatment (Lobo de Sá et al., 2021b). Prophylactic administration of resveratrol to hma IL-10^−/−^ mice starting 1 week before *C. jejuni* infection diminished the pathogen-induced colonic T and B cell responses and reduced intestinal secretion of pro-inflammatory nitric oxide, IL-6, TNF, and IFN-γ, which altogether alleviated clinical signs of acute campylobacteriosis (Heimesaat et al., 2023c).

##### 1.4.6.6 Essential oils

Essential oils are secondary metabolites of plants that can normally be extracted from various parts of the plant via hydrodistillation (Alvarez-Martínez et al., 2021). Various essential oils including those of cardamom, clove, garlic, cumin, lemon and coriander were studied for their potential use in the treatment of campylobacteriosis. Thus, treatment of microbiota-depleted and *C. jejuni*-infected IL-10^−/−^ mice with essential oils of either cardamom, clove, garlic or cumin alleviated clinical signs of acute campylobacteriosis in all cases (Bereswill et al., 2021a; Heimesaat et al., 2021b; Heimesaat et al., 2021c; Mousavi et al., 2021b). Additionally, treatment with essential oils dampened pro-inflammatory signaling in intestinal and extra-intestinal sites of infected IL-10^−/−^ mice. Interestingly, infected IL-10^−/−^ mice treated with essential oils of either cardamom, clove, garlic or cumin, resulted in significantly reduced bacterial loads in the ileum (Bereswill et al., 2021a; Heimesaat et al., 2021b; Heimesaat et al., 2021c; Mousavi et al., 2021b). Prophylactic supplementation of abiotic IL-10^−/−^ mice with essential oils of either lemon or coriander improved the clinical outcome of acute campylobacteriosis that was associated with reduced secretion of distinct pro-inflammatory molecules in mesenteric lymph nodes, kidneys and serum of infected mice (Mousavi et al., 2023b).

#### 1.4.7 Probiotics

Probiotics can play an important role in anti-*Campylobacter* strategies by their application both in farm animals by reducing bacterial loads in poultry and in humans by attenuating the infection severity (Balta et al., 2022). Probiotic bacteria, such as *Lactobacillus* and *Bifidobacterium*, show a promising potential in inhibiting *C. jejuni* colonization and infection via different mechanisms including competition for attachment sites, co-aggregation with the pathogen and production of antimicrobial compounds, such as hydrogen peroxide and/or lactic acid (Nishiyama et al., 2014). Administration of a mixture of eight different probiotic strains of *Streptococcus*, *Bifidobacterium* and *Lactobacillus*, known as VSL#3, to secondary abiotic, *C. jejuni*-infected mice, reduced pro-inflammatory (IL-6 and MCP-1) and induced anti-inflammatory (IL-10) immune responses (Ekmekciu et al., 2017). Treatment of microbiota-depleted and *C. jejuni*-infected IL-10^−/−^ mice with a commercially available probiotic Aviguard^®^ improved clinical outcome and resulted in less distinct pro-inflammatory immune responses in the intestinal and extra-intestinal sites of infected mice (Heimesaat et al., 2021d). Reassociation of secondary abiotic mice with commensal murine *Lactobacillus johnsonii* as a prophylactic regimen 14 days before *C. jejuni* infection ameliorated pathogen-induced colonic apoptosis (Bereswill et al., 2017). When the probiotic was given as a therapeutic regimen 7 days after *C. jejuni*-infection, the numbers of colonic B lymphocyte decreased, which was not the case with the prophylactic regimen. Both prophylactic and therapeutic *L. johnsonii* administration reduced ileal TNF and nitric oxide, and serum IL-6 levels, resulting in ameliorated pro-inflammatory cytokine responses in *C. jejuni*-infected mice (Bereswill et al., 2017). Interestingly, recent studies using *Caenorhabditis elegans* for infection proposed a new reliable model for a large scale screening for lactic acid bacteria antagonistic to *C. jejuni*, which can easily be translated to mouse or chicken models (Jin et al., 2021).

#### 1.4.8 Urolithin-A

Urolithins are metabolites with bioactive properties processed by gut microbiota from ellagitannins found in fruits, berries and nuts (Tow et al., 2022). Urolithin-A treatment of *C. jejuni*-infected microbiota-depleted IL-10^−/−^ mice improved clinical outcome of infected mice and slightly reduced the pathogen loads in their ileum, but not in the colon (Mousavi et al., 2021c). Additionally, urolithin-A treatment reduced intestinal and extra-intestinal pro-inflammatory immune responses in *C. jejuni*-infected IL-10^−/−^ mice, which was associated with less pronounced macroscopic and microscopic inflammatory sequelae (Mousavi et al., 2021c). The above discussed preclinical murine intervention study proposes urolithin-A as a promising treatment option for alleviating acute human campylobacteriosis.

#### 1.4.9 Vitamin C

Ascorbic acid, also known as vitamin C, is an essential micronutrient that cannot be synthesized by humans. Due to its antioxidant and immune-modulatory properties, vitamin C has a beneficial influence both on innate and adaptive immune responses against pathogens (Carr and Maggini, 2017). Several *in vitro* studies demonstrated inhibitory and even bactericidal effects of vitamin C on *C. jejuni* growth and viability (Mousavi et al., 2019a). Vitamin C administration to the secondary abiotic IL-10^−/−^ mice starting 4 days prior *C. jejuni* infection alleviated clinical symptoms of campylobacteriosis and reduced pathogen-induced apoptosis rates in colonic epithelia (Mousavi et al., 2020c). Additionally, vitamin C administration dampened pro-inflammatory immune cell responses in infected mice, as shown by lower numbers of macrophages/monocytes as well as T and B cells in their intestines (Mousavi et al., 2020c). Therefore, the preclinical intervention study using secondary abiotic IL-10^−/−^ mice as a *C. jejuni* infection model provides a new promising approach for prophylaxis and treatment of acute campylobacteriosis by vitamin C administration.

#### 1.4.10 Vitamin D

Vitamin D, which is in fact a secosteroid hormone, is known for the regulation of calcium homeostasis and gene expression associated with immunological responses against bacterial, fungal and viral pathogens (Kroner et al., 2015). RNA-Seq analysis of colonic samples obtained from *C. jejuni*-infected patients indicated that the vitamin D biosynthesis pathway is inhibited, as a putative target for the pathogen (Bücker et al., 2018). Cytotoxicity assay conducted on the intestinal HT-29/B6 cells showed that vitamin D supplementation could abolish the cytotoxic effect induced by the pathogen (Bücker et al., 2018). Treatment of secondary abiotic IL-10^−/−^ mice with synthetic 25-OH-cholecalciferol starting 4 days before infection resulted in less frequent *C. jejuni*-induced diarrhea and counteracted intestinal cell damage (Mousavi et al., 2019b). Importantly, 25-OH-cholecalciferol treatment reduced the numbers of colonic innate and adaptive immune cells along with decreased intestinal concentrations of pro-inflammatory mediators including IL-6, MCP1, and IFN-γ (Mousavi et al., 2019b). Mechanistically, it seems that *C. jejuni* targets the vitamin D receptor (VDR) pathway via VDR/retinoid X receptor (RXR) interaction, as was shown by immunofluorescence microscopy analysis (Lobo de Sá et al., 2021c). Interestingly, *C. jejuni*-induced paracellular leakiness due to mislocalization of the TJ proteins claudin-4 and occludin to cytoplasm could be inhibited by vitamin D treatment, which restored epithelial barrier integrity (Lobo de Sá et al., 2021c). Therefore, *in vitro* studies along with preclinical intervention studies provide evidence that vitamin D might constitute an effective approach to treat acute campylobacteriosis and prevent the following sequelae.

#### 1.4.11 Amalgamation of antimicrobials

Another approach assessed mixtures of several compounds in their effect on *C. jejuni*. For example, a novel phenolic antimicrobial named Auranta 3001 that mainly consisted of citrus extract, oregano extract, and grape seed extract, was found to greatly inhibit adhesion to and invasion of human epithelial cells by *C. jejuni* and *C. coli*, to reduce motility of the bacterial pathogens, and to decrease expression of genes known to be important for the function of the type VI secretion system (Sima et al., 2018). Importantly, this preparation reduced *C. jejuni* and *C. coli* loads in the cecum of infected chickens from around 8-log10 in the control groups to 2-log10 in the groups treated with Auranta 3001 after oral inoculation (Sima et al., 2018). In addition, the antimicrobial greatly reduced adherence of *C. coli* to chicken skin during slaughter by 3-log10 CFU (Balta et al., 2021a). In another study the authors combined maltodextrin, citric acid, sodium citrate, malic acid, citrus extract and olive extract and assessed the effect of the antimicrobial mixture on MDCK cells infected either with *C. jejuni*, *Salmonella enterica* or *Clostridium perfringens* (Balta et al., 2021b). This concoction significantly reduced the virulence of all three pathogens against the cell line. Specifically, as shown by measuring the transepithelial resistance (TEER), the antimicrobial restored the integrity of tight junctions, in part through increased expression of TJ proteins zonula occludens 1 (ZO-1) and occludin (Balta et al., 2021b).

### 1.5 Bacteriophages

Bacteriophages (phages) are viruses that can selectively infect specific bacterial species or strains, and therefore attract an increasing interest as they can target pathogenic bacteria without affecting beneficial microbiota and without exhibiting cytotoxicity to the host compared to some antibiotics (Baral, 2023). So far, phage treatment proved to be effective against bacterial pathogens including multidrug resistant *S. aureus*, *Pseudomonas aeruginosa* and *A. baumannii*, thus opening new strategies to overcome the rising problem of antibiotic resistance (Yang et al., 2021). Early studies by Wagenaar et al. demonstrated effectiveness of phage administration both to groups of young and pre-harvesting elder chickens, with the reduction of *C. jejuni* loads by 1–3 logs (Wagenaar et al., 2005). Later, multiple studies have shown the effectiveness of phage therapy by reducing *Campylobacter* loads in both settings *in vitro* when meat was experimentally infected and then treated, and *in vivo* when phages were administered directly to chickens (Kittler et al., 2021). While bacteriophages are considered as drugs and medicinal products in the U.S. and Europe, respectively, the lack of a legal basis for the use of phages slows down the development of phage applications (Jorda et al., 2023). Analysis of *Campylobacter* population dynamics during phage application on a commercial broiler farm demonstrated higher colonization capacity of isolates susceptible to phages in *in vitro* tests (Kittler et al., 2014). Surprisingly, bacteria susceptible to phages exhibited increased motility and gamma-glutamyl transferase activity promoting bacterial colonization (Kittler et al., 2014). Therefore, it is highly important to understand how the correlation between *C. jejuni* colonization potential and phage susceptibility can influence phage therapy in commercial broiler houses.

### 1.6 Bacteriocins

Bacteriocins (BCN) are bacteria-produced, small antimicrobial peptides that are already widely used as food preservatives, and have a promising potential in developing new treatment approaches against various microbial infections (Sugrue et al., 2024). In terms of campylobacteriosis, research has focused on preventing *C. jejuni* colonization in poultry, predominantly chickens. The first bacteriocins effective against *C. jejuni* were isolated from *Bacillus circulans* and *Paenibacillus polymyxa* and were identified as class IIa bacteriocins (Svetoch et al., 2005). Of those, BCN B 602 produced by *P. polymyxa* was tested for its ability to prevent *C. jejuni* infection in broiler chicks. While the untreated control birds showed typical colonization levels of 10^7^–10^8^ *C. jejuni* per Gram cecal content, *C. jejuni* was not detected in any of the chicks fed with B 602-treated feed prior to infection (Svetoch et al., 2005). BCN OR7 isolated from *Lactobacillus salivarus* also significantly reduced carriage of *C. jejuni* from 10^8^ CFU to approximately 20 CFU *C jejuni* per Gram of cecal material (Stern et al., 2006). Similarly, assessment of BCN B 602 and BCN OR7 in turkey chicks resulted in effective colonization with *Campylobacter coli* in the control group, but no detectable levels of *C. coli* in the BCN-treated turkey poults (Cole et al., 2006). Another bacteriocin isolated from *Lactobacillus salivarius*, BCN SMXD51, which was only analyzed *in vitro*, demonstrated antimicrobial activity against *C. jejuni* through growth reduction by two logs (Messaoudi et al., 2012). A further interesting bacteriocin, BCN E 50-52, was produced from an *Enterococcus faecium* isolate originally isolated from cecal content of commercial broilers (Svetoch et al., 2008). Initial *in vitro* experiments showed inhibition of a broad range of different pathogens, including *C. jejuni*, *Yersinia*, *Salmonella*, *Shigella*, *Morganella*, *Staphylococcus*, and *Listeria*. Treatment of *C. jejuni*-colonized broiler chicks with BCN E 50-52 reduced *C. jejuni* loads by 6-log10, but did not result in complete elimination of the pathogen. The treated broilers still carried 10^2^–10^3^ CFU *C jejuni* per Gram cecal content (Svetoch et al., 2008). Another highly active bacteriocin, enterocin E−760, was isolated from another *Enterococcus* isolate characterized as *E. durum/faecium/hirae*. BCN E−760 exhibited a broad-spectrum antimicrobial activity by inhibiting the growth of several important pathogens, including *C. jejuni*, *S. enterica*, *E. coli*, *Shigella dysenteriae* and others (Line et al., 2008). When administered as food supplement, BCN E−760 dramatically reduced the bacterial loads in young chicken by 8-log10 CFU/g cecal material, with no *C. jejuni* detected in any of the treated groups (Line et al., 2008). Further, this study showed that BCN E−760 treatment of mature broilers naturally colonized with *Campylobacter* starting 3 days before slaughter effectively reduced the bacterial loads by 5-to-6-log10 to below the detection limit of 10^2^ CFU/g (Line et al., 2008). Of all these analyzed BCNs, BCN E−760 was estimated to be the most effective. Regardless of whether the birds were treated with BCN E−760 before the experimental infection or the birds were already colonized by high *C. jejuni* numbers before BCN E−760 was administered, treatment resulted in the complete eradication of *C. jejuni* in 90% of the experiments or reduced the pathogen numbers by at least 7-log10 (Svetoch and Stern, 2010). Interestingly, when chickens colonized with *C. jejuni* were fed with BCN-supplemented chicken feed, *C. jejuni* numbers were effectively reduced. In contrast, treatment with viable bacterial cultures of the BCN-producing strain as a probiotic were ineffective in reducing *C. jejuni* in the treated birds (Stern et al., 2008).

### 1.7 Vaccine development

The development of vaccines for the prevention of *Campylobacter*-caused intestinal disease has been going on for well over 2 decades. Given that contaminated poultry meat is considered the main source of infection (Hermans et al., 2012a), large efforts have been made to prevent primary infection in broilers, on the rationale that elimination of *C. jejuni* from poultry would greatly reduce the disease burden in humans (de Zoete et al., 2007; Nothaft et al., 2016; Nothaft et al., 2021). There are several requirements that have to be met. The vaccine has to (i) be safe, (ii) elicit an efficient immune response, (iii) reliably protect against *C. jejuni* colonization, not just one strain or lineage, but the entire variability of the species, (iv) ideally, show cross-protection against *C. coli*, (v) be very easy to administer, ideally orally, and (vi) be very cheap to produce large-scale.

Similar to many other vaccines, killed entire *C. jejuni* cells were tested as vaccine candidate. Unfortunately, vaccination with this formalin-killed whole-cell vaccine failed to reduce the *C. jejuni* burden (Okamura et al., 2012). Likewise, crude cell lysates were administered, but only reduced *C. jejuni* colonization levels by about 10-fold (1-log10), and thus showed very limited effects (Annamalai et al., 2013). In addition, several protein-based, DNA-based and carbohydrate-based vaccine candidates have been tested in their ability to prevent *C. jejuni* colonization in chickens (Pumtang-On et al., 2021). Protein-based preparations included flagellin subunit FlaA (Wassenaar et al., 1993; Khoury and Meinersmann, 1995; Huang et al., 2010; Neal-McKinney et al., 2014), the multidrug efflux pump component CmeC (Jin et al., 2001; Zeng et al., 2010), the lipoproteins CjaA and CjaC (Layton et al., 2011; Laniewski et al., 2014; Wang et al., 2020), the adhesin CadF (Konkel et al., 1999b; Neal-McKinney et al., 2014), effector protein CiaB (Konkel et al., 1999a), the iron binding protein Dps (Theoret et al., 2012), and nanoparticle-encapsulated outer membrane proteins (OMPs) (Annamalai et al., 2013). Despite the induction of promising target-specific antibody production in most cases, the elicited immune response was associated with problems. The vaccination either provided only limited protection, lacked cross-protection or targeted epitopes with high genetic variability, or resulted in varying protection levels that depended on how the vaccine was administered, or did not sufficiently prevent *C. jejuni* colonization (Khoury and Meinersmann, 1995; Zeng et al., 2010; Layton et al., 2011; Theoret et al., 2012; Annamalai et al., 2013; Neal-McKinney et al., 2014). Another approach identified several *C. jejuni* OMPs as possible vaccine candidates *in silico* (Meunier et al., 2016) and used DNA-based preparations of hemolysin activation/secretion protein or flagellin protein FlgL to vaccinate chickens, but those showed either no effect or provided limited reduction of *C. jejuni* numbers (Meunier et al., 2017; Meunier et al., 2018). A recent review of completed vaccine trials summarized the data from several studies and listed the vaccine efficacy in terms of reduction of *C. jejuni* colonization (Pumtang-On et al., 2021). This overview concluded that a vaccine consisting of 125 µg crude cell lysate supplemented with OMPs covered in nanoparticles showed the highest efficacy against *C. jejuni* colonization. *C. jejuni* loads were reduced by 5.7-log10 CFU per Gram of fecal content, and thus showed 90% efficacy (Annamalai et al., 2013). However, while the OMP vaccine reduced *C. jejuni* cecal loads below the detection limit of 10 CFU per ml cecal content in all vaccinated chickens, the vaccine was only effective when administered subcutaneously. After oral vaccination, 63% of the tested chickens were still positive for *C. jejuni* (Annamalai et al., 2013). Given, that only subcutaneous vaccine administration provided significant protection against *C. jejuni* infection, this vaccine may not be suitable for large-scale use in broiler farms. Another vaccine strategy that also resulted in efficient protection of chickens against *C. jejuni* consisted of oral immunization of chickens with a live, avirulent *S. enterica* sv. Typhimurium strain expressing *C. jejuni* gene *cjaA* (cj0982c). The vaccinated birds not only developed serum IgG and mucosal IgA antibody responses against the *C. jejuni* CjaA membrane protein (and also *Salmonella* OMPs), but importantly greatly reduced the ability of *C. jejuni* to colonize the chicken intestinal tract, which was estimated to 89% efficacy (Wyszynska et al., 2004; Laniewski et al., 2014).

Other vaccine development efforts focused on *C. jejuni* carbohydrates (Keo et al., 2011; Hodgins et al., 2015; Nothaft et al., 2016). Vaccination of chickens with a capsular polysaccharide - diphtheria toxoid conjugated vaccine, which was administered subcutaneously, only resulted in a 0.6-log10 reduction in *C. jejuni* numbers compared to the non-vaccinated control birds (Hodgins et al., 2015). Based on research showing that *C. jejuni* express multiple different surface carbohydrates including O-linked and N-linked glycans (Szymanski et al., 2003) and that N-glycosylation of surface proteins promotes *C. jejuni* fitness (Szymanski et al., 2002; Alemka et al., 2013), N-glycan was evaluated as a potential vaccine candidate to control *C. jejuni* in chickens (Nothaft et al., 2016; Nothaft et al., 2021). N-glycan, that is common to all *C. jejuni* and *C. coli* strains, consists of a heptasaccharide composed of five units of N-acetylgalactosamine, one unit glucose and one unit diacetamido-trideoxy-D-glucopyranose (Young et al., 2002). Chickens vaccinated by oral administration of 10^8^ live *E. coli* cells expressing *C. jejuni* N-glycan instead of *E. coli* O-antigen showed up to 8-log10 reduction in *C. jejuni* in cecal content (Nothaft et al., 2016). Unfortunately, not all birds responded to vaccination, which was associated with differences in the intestinal microbiota (Nothaft et al., 2017), a problem that had also affected other vaccine trials. Increased levels of Clostridia, specifically OTUs classified as *Clostridium* spp., Ruminococcaceae and Lachnospiraceae, were found to be beneficial for vaccine efficacy (Nothaft et al., 2021), and co-administration of the live N-glycan-based vaccine with the Clostridiales bacterium *Anaerosporobacter mobilis* that was identified in good vaccine responder chickens or with the probiotic bacterium *Lactobacillus reuteri* significantly improved vaccine performance (Nothaft et al., 2017). Thus, vaccine development and improvement will very likely continue.

### 1.8 The “One World–One Health” strategy

The “One World-One Health” (or short “One Health”) concept originated from the “One Medicine” approach, which suggested the establishment of an interdisciplinary cooperative research alliance of human and veterinary medicine to better control specific diseases, as originally designed by Calvin W. Schwabe about 40 years ago (Schwabe, 1984). The rationale of this initiative is to obtain a more comprehensive understanding of human and animal health and disease. In this context, the Wildlife Conservation Society has published the “Manhattan principles”, synopsizing practical methodologies and common standards in this concept (One World One Health, 2020). In addition, the so-called “Tripartite Concept Note” has been published, which represents a strategic paper for a partnership between the World Health Organization (WHO), the World Organization for Animal Health (WOAH), and the Food and Agriculture Organization of the United States (2025), focusing on the health risks during interactions of humans with domestic animals, wildlife and natural ecosystems, including the fight against antimicrobial resistances and specific microbial diseases. These ideas have been further optimized in recent years as a promising strategy for better control of zoonotic infectious diseases, additionally involving environmental health (One World One Health, 2021; Pitt and Gunn, 2024; Zhang et al., 2024). As a result, this concept was recently expanded to the “Quadripartite collaboration on One Health” that was initiated by the WHO, the WOAH, the FAO, and the United Nations Environment Programme (UNEP) to sustainably balance and optimize the health of humans, animals, plants and the environment (FAO, UNEP, WHO and WOAH, 2022). The One Health concept is particularly important for *Campylobacter* as a major zoonotic pathogen in the bacterial kingdom (Heimesaat M. M. et al., 2021). An example for its implementation is the PAC-Campy consortium that was launched under the direction of the Federal Ministry of Education and Research (Pac-Campy, 2023). This association is part of the National Research Network for “Zoonotic Infectious Diseases” and investigated *Campylobacter* infections in Germany from 2017–2023. The increasing number of reported cases of campylobacteriosis in humans emphasized the need to better understand the *Campylobacter* population genetics, the molecular basis of the infection, and to develop new strategies for the prevention and control of the pathogen and treatment of the disease (Lobo de Sá et al., 2021a; Golz and Stingl, 2021; Püning et al., 2021; Szott and Friese, 2021; Tegtmeyer et al., 2021; Linz et al., 2023). The overall objective of the PAC-Campy consortium was to reduce the contamination of food with *Campylobacter* and the incidence of these infections in humans. Poultry for fattening is a major natural reservoir for the pathogen, and there is a clear link between the presence of *Campylobacter* in broilers and *Campylobacter* infections in humans that can be traced back to the consumption of contaminated chicken meat (Ben Romdhane and Merle, 2021; Epping et al., 2021). Therefore, the particular aim of the network was to reduce the contamination of poultry meat with *Campylobacter*, and thus the number of infections in humans through intensive co-operation between all partners. To this end, possible intervention strategies along the production chain for poultry food products are being analyzed in terms of their efficiency and practical feasibility (McKenna et al., 2020; Pangga et al., 2025). Based on the data obtained, the research network has developed management strategies and intervention models such as bacteriophages and tested them under field conditions (Alter and Reich, 2021; Kittler et al., 2021). In addition, PAC-Campy provided the public health service and industry with efficient intervention measures (BMBF, 2021; Backert, 2021; Pac-Campy, 2023). In complementary work, the research network developed infection models in mice tested with numerous therapeutic compounds discussed above in order to establish innovative approaches for combating infections in humans and investigating the course of outbreaks (Mousavi et al., 2021a; Foote et al., 2023; Heimesaat et al., 2023b; Omarova et al., 2023).

## 2 Conclusion and perspectives

*C. jejuni* is a major globally distributed zoonotic pathogen with a high infectivity in humans. The pathogen cannot yet be effectively eliminated from the poultry food chain and represents one of the four key sources of diarrheal disease worldwide (WHO, 2020; WHO, 2024). This high incidence of the disease and the potential of late complications brands this pathogen as a serious threat for mankind both from medical and socio-economic viewpoints. Therefore, “One World-One Health”-based control processes and intervention strategies have been recently developed in order to minimize the occurrence of the bacteria in livestock and reduce contamination during food production. For example, new technological decontamination measures should help to improve hygiene at the fattening, slaughter and processing levels. At the same time, general awareness of foodborne pathogens such as *C. jejuni* must be further promoted among the human population through public information campaigns. The focus here should be on kitchen hygiene during processing of raw poultry meat products, which can even distribute the pathogen to other food. In addition, much progress has been made in recent years to unravel in detail the molecular mechanisms of *C. jejuni* pathogenicity, which are now also utilized to develop new therapeutics, phage protocols and vaccines against campylobacteriosis. Future studies should therefore intensify the search for and the investigation of novel anti-bacterial and anti-inflammatory compounds to reduce the *C. jejuni* load in the gut, and to impede associated inflammation and apoptosis, which in turn may not only diminish diarrhea, but also help to protect against secondary diseases caused by the pathogen such as GBS, MFS, RS or various chronic inflammatory processes in humans.
